# An evaluation of a data linkage training workshop for research ethics committees

**DOI:** 10.1186/s12910-015-0007-y

**Published:** 2015-03-04

**Authors:** Kate M Tan, Felicity S Flack, Natasha L Bear, Judy A Allen

**Affiliations:** Telethon Kids Institute, The University of Western Australia, PO Box 855, West Perth, WA 6872 Australia; Telethon Kids Institute, The University of Western Australia, PO Box 855, West Perth, WA 6872 Australia; Telethon Kids Institute, The University of Western Australia, PO Box 855, West Perth, WA 6872 Australia; Faculty of Law, University of Western Australia, 35 Stirling Highway Crawley, WA 6009 Perth, Australia

**Keywords:** Data linkage, Record linkage, Ethics, Law, Training

## Abstract

**Background:**

In Australia research projects proposing the use of linked data require approval by a Human Research Ethics Committee (HREC). A sound evaluation of the ethical issues involved requires understanding of the basic mechanics of data linkage, the associated benefits and risks, and the legal context in which it occurs. The rapidly increasing number of research projects utilising linked data in Australia has led to an urgent need for enhanced capacity of HRECs to review research applications involving this emerging research methodology. The training described in this article was designed to respond to an identified need among the data linkage units in the Australian Population Health Research Network (PHRN) and HREC members in Australia.

**Methods:**

Five one-day face to face workshops were delivered in the study period to a total of 98 participants. Participants in the workshops represented all six categories of HREC membership composition listed in the National Health and Medical Research Centres’ (NHMRC) National Statement on Ethical Conduct in Human Research. Participants were assessed at three time points, prior to the training (T1), immediately after the training (T2) and 8 to 17 months after the training (T3).

**Results:**

Ninety participants completed the pre and post questionnaires; 58 of them completed the deferred questionnaire. Participants reported significant improvements in levels of knowledge, understanding and skills in each of the eight areas evaluated. The training was beneficial for those with prior experience in the area of ethics and data linkage as well as those with no prior exposure.

**Conclusions:**

Our preliminary work in this area demonstrates that the provision of intensive face to face ethics training in data linkage is feasible and has a significant impact on participant’s confidence in reviewing HREC applications.

## Background

Data linkage is now a vital and rapidly expanding part of Australia’s research and policy development activity. Data linkage is a method of bringing together information from different sources, but relating to the same individual or event into a single file [1]. This can be done in a way that minimises risks to individual privacy [2]. Data linkage enables researchers to conduct valuable whole of population research to assess the safety, quality and costs of health care and explore the causal pathways to ill health. For example a study by Mathews JD et al. linked data from the Australian Medical Benefits Scheme, the Australian Cancer Database and the National Death Index to determine the cancer risk in children and adolescents following diagnostic computed tomography scans [3].

Prior to 2009 Western Australia (WA) and New South Wales (NSW) were the only Australian states with data linkage units [4]. In 2009 the Australian Government invested heavily in data linkage, recognising the central role it will play in Australia’s research environment in the future [5]. This investment saw the establishment of data linkage units representing each of the remaining states and territories as well as two national data linkage units. The Population Health Research Network (PHRN) is a network of these data linkage units across Australia. This expansion of data linkage infrastructure is expected to increase the attractiveness of linked data to many researchers as the availability and accessibility of data collections improves [6].

Data linkage requires the use of personal information to make the initial link between data collections [2,6-8]. This is usually done without consent and therefore significant legal and ethical issues arise [7-9]. In Australia, research projects using linked data must be approved by three groups: the data linkage unit; all participating data custodians; and a Human Research Ethics Committee (HREC). A national scoping study, which assessed the needs of these stakeholders, was conducted by the PHRN in 2011. The study indicated concerns that HREC members were lacking in understanding of the process of data linkage. Findings from this study identified training for HREC members as a priority [10].

The National Health and Medical Research Council (NHMRC) ^a^ mandates that all HREC members attend continuing education or training programs in research ethics at least every three years [11]. With over 1,600 members nationally [12], this requirement generates a large demand for human research ethics training.

Data linkage research can provide enormous benefit to the community [8,9,13], however public support for the use of linked data is contingent on the protection of privacy and confidentiality [2,13]. Concerns about privacy and confidentiality in relation to the use of linked data threaten to stifle its progress [6,8]. The future of data linkage depends on the conduct of high quality research projects, the protection of data and ethical conduct of researchers [14,15]. Banos et al. [16] argues that a HREC’s role is not only to provide protection of research participants, but also to provide an “adequate public guarantee in this regard”. Understanding and awareness of data linkage is generally low in the community and if HRECs are to meet this dual role in relation to data linkage projects they need to have sufficient technical understanding to assess the risks to privacy and confidentiality and to reflect on the ethical issues.

It is well recognised that education efforts are important and can improve the knowledge, attitudes and skills of HREC members [15-20]. Despite the demand for general ethics training in Australia, availability remains ad hoc and limited, particularly for face to face workshops. Several web-based introductory training programs are available, notably those provided by Monash University, Macquarie University and more recently the University of Wollongong. Specialised training in particular research methodologies is beyond the scope of these introductory programs.

This paper describes the development and delivery of a face to face training program designed for people involved with HRECs in Australia and provides the results of a three phase participant evaluation of the training workshop.

The purpose of this project was to determine:

- the demand for training in ethics and data linkage for HREC members;
- the optimal format for the training; and
- whether the training could increase participants’ confidence and ability to make informed decisions when reviewing data linkage applications.

The evaluation project was reviewed and approved by the Sir Charles Gairdner Group Human Research Ethics Committee in accordance with the requirements of the NHMRC National Statement on Ethical Conduct in Human Research.

## Methods

### Stage 1: National scoping study

A national scoping study was conducted by the PHRN in 2011 to determine the training needs and priorities of PHRN data linkage units across Australia. The findings revealed a need for training of a number of groups including data custodians and researchers, however, training for HREC members in the area of data linkage was identified as a priority by a number of the data linkage units consulted [10]. The respondents in the scoping study took the view that members of HRECs did not have a sufficient understanding of the processes of data linkage to equip them to engage with the ethical issues and that there was a need for specialised training. To confirm and validate these findings, the PHRN surveyed representative HRECs from across Australia as the next phase of the national scoping study. The PHRN was particularly interested in understanding the demand for training in ethics and data linkage for HREC members and the optimal format for any training provision. Twenty three of the 30 HRECs contacted participated in the survey (response rate 77%). Of those who responded to the survey, 87% were interested in receiving training specific to data linkage with face to face training the preferred mode of delivery. Based on the survey results and research findings supporting face to face training as an effective mode of delivery for ethics training [14,20,21] a one day, face to face workshop was developed.

### Stage 2: Development of training program

The aim of the training was to support existing HRECs to effectively review research applications involving linked data. The training sought to increase participants’ (HREC members’) confidence and ability to make informed decisions when reviewing data linkage applications. The technical content of the training materials reflected the core values from the NHMRC National Statement [11] including; respect for human beings, research merit and integrity, justice, and beneficence. The training content was informed by the scoping study findings and sought to provide participants with the necessary foundations of data linkage methodology and the skills required to assess the principal ethical concerns in the context of data linkage. Particular attention was given to evaluating the benefits of data linkage and assessing and minimising the risks to privacy. The ethical issues surrounding a waiver of participant consent were explored in light of the relevant NHMRC National Statement principles and the guidelines in the applicable privacy legislation.

Staff of the PHRN Program Office developed training materials in consultation with a reference group of experts within Australia who specialise in the areas of data linkage, ethics, privacy, law and consumer participation. The handout booklet produced for the workshop included PowerPoint slides, lecture notes and activities and was initially developed to accommodate for those learning styles that find such resources beneficial and to improve the sustainability of participants’ learning. The training materials were developed in line with the principles of the Adult Learning Theory and Skills Approach [22] with an emphasis on high levels of participant engagement and practice activities [14,16-18,20-23]. The training included didactic presentations, group work, case-studies and problem solving activities to achieve program objectives [18,24,25].

Training methods were chosen to optimise learning and skill acquisition. One of these methods was the use of case studies based on local examples. The use of local examples is thought to enhance learners’ abilities to identify and address ethical dilemmas more effectively than cases that seem unlikely or irrelevant to their situation [18,20,24]. In addition, to accommodate the variations in legislation across the states and territories and the data collections available for linkage, training materials were tailored for each state and territory.

Two pilot workshops were conducted prior to the national roll-out of the training to determine program timing, content load, relevance of information and to trial activities [21]. Changes were made to a number of the activities to improve clarity and adjust timings. The pilot workshops were also used to determine the optimal method for involving staff from the local data linkage units [15]. For the first pilot a panel discussion was conducted in the last session of the day. Panel members included data linkage unit staff, Director of the PHRN, a researcher and a consumer representative. Alternatively, two staff members from the local data linkage unit attended the second pilot for the full day to answer participants’ technical questions pertaining to data linkage throughout the day. The participation of the local data linkage unit was deemed positive and their participation was incorporated into the training program.

### Stage 3: Recruitment of participants for the training program

The training was targeted at all members of HRECs registered with the NHMRC and ethics office staff. The inclusion of ethics office staff at the training with HREC members was regarded as important because they are the first point of contact for researchers [26].

Chairs and ethics office staff of each HREC within the respective states were contacted and invited to register members of their committee for the training. Attendance at the training was voluntary and incurred a moderate fee. The fee was calculated to cover the cost of running the workshop including venue hire, catering, travel and accommodation for the facilitators. The majority of the attendees had their fees paid for by their institution but some individuals paid their own fees.

### Stage 4: Delivery of the training program

Five workshops were held between March 2011 and November 2012 in capital cities in South Australia (SA) (1), New South Wales (NSW) (2), Australian Capital Territory (ACT) (1) and Tasmania (1). The workshops were conducted by two senior staff of the PHRN. The same facilitators delivered all five of the trainings evaluated in this study. Each workshop was one full day in length. Staff from the local data linkage unit were present for the full day to provide local specific technical input as required.

The training was provided to small groups with participant numbers capped at 24. This restriction was implemented in response to evidence of increased effectiveness with smaller groups [16]. A total of 98 participants attended the five training workshops between March 2011 and November 2012. This equated to 90% (n = 109) of those who registered for the training. The majority of participants (73%; n = 72) were women. This is not necessarily reflective of HREC membership generally. The NHMRC National Statement requirement is that as far as possible a HREC should comprise of equal numbers of men and women [11]. Participants represented all six categories of membership composition listed in the NHMRC National Statement [11].^b^ Ethics office staff, lay people and research representatives were the most strongly represented categories.

At the workshop all participants were given a handout booklet of course materials containing theory, activities, and the PowerPoint presentation utilised at the workshop. Certificates of completion were issued to those participants who completed the full day training.

### Stage 5: Training evaluation

The purpose of the evaluation was to determine whether the training could increase participants’ confidence and ability to make informed decisions when reviewing data linkage applications.

Participants who attended the full day of training were asked to complete an anonymous survey evaluating various aspects of the training program. Consent was evidenced by completion of the survey. Participants were assessed three times: prior to the training (T1), immediately after (T2) and eight to 17 months later (T3). The first two assessments were carried out at the training site and numeric identifiers were used allowing pre and post-test to be paired. The third assessment (T3) was conducted over the internet using Survey Monkey. Training participants who provided their contact details were sent an email inviting them to participate in the deferred web-based survey (T3). Participants were given four weeks to respond to the survey. A reminder email was also sent to those participants who had not completed the survey within two weeks. The deferred web-based survey was also anonymous, however as no numeric identifiers were used, data from the third assessment could not be linked with the first two assessments at a participant level.

Pre and post training surveys included eight impact statements measuring participants’ self-reported levels of knowledge, perceived levels of skill development and confidence. The respondents indicated their levels of knowledge using a Likert scale with grades from one to five: (1 = very low; 2 = low; 3 = average; 4 = good; 5 = very good).

The deferred web-based survey (T3) was carried out among those individuals who completed the full day training and who provided contact details. The main purpose of the 14-question survey was to determine the applicability of the materials, knowledge and skills obtained during the training, and to get participants perspectives on whether any improvements had taken place in relation to their confidence and ability to review data linkage ethics applications as a result of the training.

### Stage 6: Statistical analysis

Survey results were described using frequencies and percentages. For questions scored using a Likert scale the median and interquartile range were reported, as the Likert scale is considered ordinal. Comparisons pre to post training were performed using the Wilcoxon Signed Rank test. Further analysis was performed comparing those participants with and without experience in reviewing data linkage applications. For these two groups, between group and within group comparisons were implemented using Mann Whitney U and Wilcoxon signed rank respectively.

All statistical analysis was performed using Stata10 Version 10.1 (StatCorp, Texas).

## Results

### Survey participants

A total of 75 (81%) participants completed both the pre- (T1) and post- (T2) test questionnaires. Participants arriving late to the training or leaving shortly prior to the end of training explained the majority of missing data. Of those 84 participants who were emailed the web-based survey (T3), 57% completed the deferred web-based survey (n = 48) which is above the reported average response rates for online surveys [27].

Prior to the training, participants were asked to indicate previous training, experience and confidence in reviewing data linkage applications. Of the 85 respondents who completed the pre-test questionnaire (T1), 12% (n = 10) had previously received training on ethics or data linkage and 63% had previously reviewed a data linkage application. Of those who had reviewed a data linkage application 50% indicated that they felt confident, 33% not confident and 17% somewhat confident. For those who had never reviewed a data linkage application the majority (77%) indicated that they did not feel confident.

### Pre and post workshop (T1 and T2) responses

Table 1 displays the pre-to-post workshop responses. Overall there was a significant increase in participants self-evaluation of knowledge and understanding for all eight impact statements following the training (p < 0.001).

**Table 1:** **Workshop questionnaire – pre and post workshop comparisons (median and inter-quartile range) using a Likert scale with grades from one to five (1 = very low; 2 = low; 3 = average; 4 = good; 5 = very good)**

**WORKSHOP IMPACT FOR YOU**	**Pre test median (IQR)**	**Post test median (IQR)**	p value*
Please indicate the effect this workshop has had on:			
1. Your understanding of the data linkage process	3(2–4)	4 (4–5)	<0.001
2. Your knowledge of data collections available for linkage in [the relevant jurisdiction]	3 (1–4)	4 (3–4)	<0.001
3. Your awareness of the history of data linkage	2 (1–3)	4 (4–5)	<0.001
4. Your understanding of the Population Health Research Network	2 (1–3)	4 (3–4)	<0.001
5. Your awareness of the benefits of data linkage	4 (3–4)	4 (4–5)	<0.001
6. Your knowledge of strategies to minimise and manage risks associated with data linkage	3 (2–4)	4 (4–5)	<0.001
7. Your understanding of the legal framework applicable to data linkage	2 (1–3)	4 (3–4)	<0.001
8. Your ability to consider a waiver of consent for data linkage projects	3 (2–3)	4 (4–4)	<0.001

*pre to post comparison using Wilcoxon Signed Rank Test.

Results from one of the training workshops showed a substantial number of participants reporting a decrease in knowledge/understanding (29%) in at least one of the eight impact statements following the training. This group recorded at least one reduction in score pre-to post-testing in all but one question, with question 4 (understanding of the PHRN) receiving no reduction in scores. These findings were inconsistent with the overall findings of the study and were drawn from the second of the NSW workshops. This workshop was different to other workshops which may explain the reported decreases. This workshop had a much wider range of prior knowledge among participants than seen in other groups. It is possible that exposure to highly experienced and skilled members may have resulted in some participants adjusting their view of their knowledge. This is consistent with feedback from participants during the training that they had reframed their perceived level of understanding as they gained an appreciation of the complexity of the area. This training workshop had more participants than other workshops which may have impacted on their learning experience.

Prior to the training, participants who had never reviewed an ethics application proposing the use of linked data had overall a lower reported understanding of data linkage processes, less awareness of the benefits of data linkage, lower self-reported knowledge of strategies to minimize risk and believed they had a lower ability to consider a waiver of consent for data linkage compared to those who had previously reviewed data linkage applications (median scores 2 vs 3 respectively for questions 1, 6 and 8 and median score 3 vs 4 for question 5, all p < 0.05). There did not appear to be any difference between those with or without prior experience in relation to understanding the legal framework, with both groups reporting a median of 2 indicating a low level of understanding in this area prior to training. At the completion of the training both groups reported an improvement in awareness and understanding of data linkage processes, benefits, risks, legal framework and consent waivers. Following the training there was no difference in all questions between those who had or had not previously reviewed an application (p > 0.05). Table 2 highlights the relationships between group and within groups for participants who had some or no experience in reviewing a data linkage application.

**Table 2:** **The impact of prior review experience on training outcomes: a comparison**

		**Ever reviewed a data linkage application**		
		**No**	**Yes**	p value*
		**Median (IQR)**	**Median (IQR)**	**(Between group comparison)**
1. Your understanding of the data linkage process	Pre	2 (1–2)	3 (2–4)	0.001
	Post	4 (4–5)	4 (4–5)	0.761
5. Your awareness of the benefits of data linkage	Pre	3 (2–4)	4 (3–5)	0.012
	Post	4 (4–5)	4 (4–5)	0.965
6. Your knowledge of strategies to minimise and manage risks associated with data linkage	Pre	2 (1–3)	3 (2–4)	0.046
	Post	4 (4–5)	4 (4–5)	0.883
7. Your understanding of the legal framework applicable to data linkage	Pre	2 (1–3)	2 (2–3)	0.090
	Post	4 (3–4)	4 (3–4)	0.119
8. Your ability to consider a waiver of consent for data linkage projects	Pre	2 (1–3)	3 (2–4)	0.007
	Post	4 (4–4)	4 (4–5)	0.177

*between group comparison (those who had previously reviewed a data linkage application vs those who had not) using Mann Whitney *U* test.

**Figure 1:**
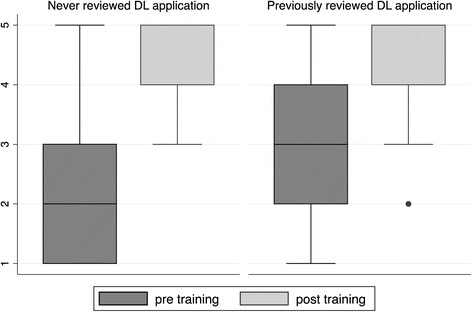
**Relationship between understanding of data linkage processes and previous experience in reviewing data linkage applications.**

Figure 1 shows that prior to the training the two groups reported different levels of knowledge, however post training the distributions are similar indicating a comparable level of understanding. It also demonstrates that both groups reported improvements following the training.

The post-test questionnaire asked participants to rate, using a 5-point Likert scale (1 = not at all to 5 = strongly agree), workshop structure and training delivery. As shown in Table 3, the workshop was highly regarded overall by participants, in all aspects of workshop structure and facilitation.

**Table 3:** **Participant ratings of workshop structure and facilitation**

**Workshop structure and facilitation**	**Median**	**Mean**
The workshop objectives were clear to me	5	4.4
The topics covered were relevant to my work	5	4.4
Frequency and lengths of breaks were good	4	4.6
Training handouts were well presented and easy to follow	5	4.7
The length of the training was appropriate	5	4.6
The trainers provided opportunities to practice skills	5	4.6
The trainers were well prepared	5	4.6
The trainers knew the subject material well	5	4.7
The trainers were enthusiastic	5	4.7
The trainers related well to me	5	4.6
The trainers were clear in their presentation	5	4.6

### Deferred survey (T3) responses

Of those who responded to the deferred web-based survey (n = 48), 77% were women (n = 37) which is consistent with the gender response rate for T1 and T2. The categories most strongly represented were researchers, health professionals and lay people (32%, 24% and 18% respectively). Respondents represented each of the five workshops conducted.

Of those who responded, 69% had completed a review of a linked data research application since their training. The handout booklet was well utilised post training with 90% of respondents referring to the handout booklet in their review of data linkage applications post training. All of the respondents who utilised the handout booklet indicated that they found it useful.

The training was effective in improving participant’s self-reported understanding of the data linkage process. The majority (91%) of respondents indicated that the training improved their understanding either “very much” (56%; n = 17) or “moderately” (35%; n = 11).

Confidence in reviewing data linkage applications improved as a result of the training for 87% of respondents. Two of the four respondents who indicated that the training had no impact on their confidence noted in the comments section of the survey that they had high levels of knowledge in the area prior to the training. One of these respondents noted:

“The workshop was excellent. I had a fairly high level knowledge before I started hence some of my responses. I know it has had a significant impact on a number of other members who attended.”

There were six impact statements included in the deferred web-based survey. Participants were asked to rate the impact of the training on a 5-point Likert scale (5 = very high; 4 high; 3 moderate; 2 low; and 1 no impact). In interpreting six impact statements from the deferred web-based survey, we considered a rating of moderate to very high as a positive impact. The deferred web-based survey revealed that the training had a positive impact on participant’s self-reported ability to review the NHMRC National Statement’s core values, assess risks (*M* = 4), assess benefits (*M* = 3), assess researcher’s strategies to minimise and manage risks (*M* = 4), and their understanding of the legal framework in relation to data linkage applications (*M* = 3).

All participants indicated that they would recommend the training to other HREC members.

## Discussion

### Is there a need/demand for training in data linkage for HREC members?

Our study found that there is a strong demand for training in the area of ethics and data linkage from members of HRECs in Australia. Currently in Australia institutions that convene HRECs have an obligation to provide their members with training but there is no institution that has explicit responsibility for providing training to HREC members generally and there are few face to face training opportunities available.

Of the HRECs who participated in the national scoping study conducted by the PHRN, 87% indicated that they were interested in receiving ethics training specific to data linkage. This initial interest in training was followed up by high demand for places in the training workshops and high attendance rates, with 90% of those who registered attending the full day.

The demand for the training the PHRN offered reflects an unmet need for ethics training in Australia and the recognition of the increasing presence of data linkage in Australia’s research environment. This was supported by pre-test data which showed that two thirds of participants had previously reviewed a data linkage application but only half of those had felt confident in doing so.

This was the first ethics training program to be offered in Australia which focused specifically on the legal and ethical issues surrounding the use of linked data and this is thought to be a strong contributing factor to the high demand for the training. Additional factors may also have contributed to the high demand and attendance levels. Registration fees were low due to facilitator costs being provided by the PHRN in-kind and the full day program was run offsite in each state and territory which encouraged participants to attend for the whole day [16,17].

It is noteworthy that prior to the training only half of those who had previously reviewed data linkage ethics applications felt confident to do so. This reflects the challenge that generalist ethics review boards face in providing effective review of all kinds of research. We may be expecting too much of ethics committees if we require them to operate across the whole field of research. It is difficult to analyse the ethical issues involved in a research project without a basic understanding of the procedures involved in the methodology [28]. These results support the need for training of members of ethics committees which focuses on particular methodologies (e.g. data linkage, genomics). Our study also highlights the need to consider whether ethics committees which specialise in particular kinds of research may provide a stronger system of ethical review.

### What is the optimal format for face to face ethics training in data linkage?

A one day, face to face workshop was chosen as the training format following a review of the available literature and after consultation with HRECs members. Face to face training was the preferred mode of delivery for 87% of the HRECs which responded to the survey on training needs which was part of the national scoping study.

This study was not designed to compare the value of online versus face to face training, rather this study sought to specifically determine the optimal format for face to face training.

Our research showed that not only is there a strong demand for face to face training opportunities for HREC members, it also provided the expected benefits as described in the literature [14,18,20,29,30]. Face to face training provided participants with a unique opportunity to gain valuable insight into their peers different points of view and approaches and required participants to articulate their ethical reasoning processes [23,29]. The training activities simulated the interactions among ethics committee members in their deliberations, allowing participants to apply the skills and knowledge acquired during the training [14]. These elements were critical in consolidating the participants’ skill development and improving participants’ confidence and understanding of data linkage [25].

Review of participation rates and assessment results supports the notion that intensive face to face training is feasible, effective and is worth maintaining. The sustainability and geographical availability of training is dependent on appropriate funding. In this case the development and delivery of the training were supported by the PHRN with fees set for recovery of logistical costs.

The format and content of the workshop was developed after extensive consultation with HREC members and experts from all relevant disciplines. This inclusive process resulted in the development of a high quality and relevant training program that resulted in positive outcomes for both participants and data linkage unit staff.

The inclusion of local data linkage staff in the training was seen as essential to ensure the highest quality of training provision and to enable a local and relevant focus [15,29]. The two pilot workshops held in WA were used to obtain feedback on course content and structure as well as determine the preferred method to involve data linkage unit staff. The presence of data linkage staff throughout the whole workshop was better utilised and valued by participants than the panel discussion. Feedback from the workshops indicated that including data linkage unit staff not only provided further depth to the training, but also had benefits for HREC members and data linkage unit staff. The data linkage unit staff stated that observing the training gave them invaluable insight into how HREC members viewed data linkage and some of the concerns they had pertaining to data linkage. It also established a link between data linkage unit staff and HREC members and provided an important foundation for a working relationship between the two groups. It is unlikely that these benefits of personal interaction could have been achieved had the training been delivered online. These findings are particularly relevant in an environment where there is a significant movement towards online training.

The workshops were delivered by two trainers with expertise in ethics and law who were able to provide a responsive approach which increased participants’ opportunity to receive guidance, feedback and reinforcement [22,29,31]. Choosing multiple facilitators with a complementary set of expertise enriched the training. Other training evaluations have also reported the benefits of using multiple facilitators, particularly where disciplines reflected those of the participants, thus allowing them to act as role models [16,31].

The second and third evaluations indicated that the handout booklet was a valued resource for most participants. Findings from T2 showed that participants found the handout booklet very useful during the training. Furthermore findings from T3 indicated that 90% of respondents who reviewed data linkage applications following the training reported using the handout booklet in their review, with all stating that it was useful. Other studies have confirmed that the development of detailed handout materials that can be utilised post training has enduring benefits [21].

The number of participants at a workshop impacted on participants’ self-reported learning. Due to the strong demand for the training in NSW, 27 participants were registered. Results from the post-test (T2) and deferred web-based survey (T3) showed that expanding group numbers compromised some of principles the Adult Learning Theory and Skills Approach. Notably the opportunity for feedback, guidance and reinforcement effected participants’ experiences. Bylund et al. [22] notes the critical role of feedback in the process of developing new skills as part of the learning process. The impact of increasing participant numbers also had a direct impact on learning outcomes. Participants who attended trainings of larger group sizes reported lower mean differences than those who were in smaller groups.

### Can a training workshop increase participants’ confidence and ability to make informed decisions when reviewing data linkage applications?

The majority of participants (87%) who completed the deferred survey (T3) indicated that they were more confident about their ability to review data linkage applications as a result of the training workshop. It is noteworthy that, even though those who had previously reviewed an application started with higher scores (T1), they still showed significant improvement post training (T2). This finding is similar to those evidenced in previous studies and may be attributable to the active learning methods adopted and facilitation by experienced multidisciplinary trainers [17].

The evaluation conducted immediately after the training (T2) could be considered a reactionary evaluation which was biased by participants’ enjoyment of the day. Therefore it was considered important to conduct a third evaluation (T3), once participants had had the opportunity to apply their knowledge and skills in reviewing applications to use linked data, to determine whether they still felt confident some months after the training. The majority of the respondents (87%) to the web-based survey, conducted 8–17 months after the training, indicated improved confidence in their abilities. This was an extremely encouraging result. Whilst this provides some insight into the sustainability of the learnings, further research is required to measure skills and provide more specific enquiry. This training was designed for members of HRECs who regularly review data linkage projects. The sustainability of the results is more likely to be significant for those who have the opportunity to practice the skills regularly.

## Conclusions

Specialised training for HRECs in this emerging area will help ensure the ethical conduct of research and is an essential component in the development of data linkage strategies in Australia if its potential is to be fully realised. Our preliminary work in this area demonstrates that the provision of intensive face to face ethics training is feasible and has a significant impact on participant’s confidence in reviewing HREC applications. The authors think the conclusions relating to the optimal format for face to face training are generalizable, particularly to training focusing on specialist areas both in Australia and internationally.

### Limitations

There were limitations to this study. Firstly, the study measured participants’ self-perceived levels of knowledge and skills. To determine whether the training contributes to better research ethics review, and ultimately to better protection of research participants, it would be necessary to conduct further research. Such research should assess whether the training had an actual impact on participants’ skills and include a follow up of the scoping study to determine if the concerns regarding HRECs capacity to review data linkage projects were addressed as a result of the training. This could only be achieved through pre and post training measurements of participants’ understanding of ethical principles and the legal framework pertaining to data linkage [29] and follow up interviews with DLU staff.

Second, the decision to conduct a deferred web-based survey (T3) was made after all of the trainings had been delivered. This had an impact on the study as T3 could not be linked to T1 and T2.

Finally, the timing of the deferred web-based survey (T3) varied between training groups. Due to funding limitations, the time between the delivery of the Tasmanian training and the deferred survey was too short for many of the participants to have had the opportunity to review a research application proposing the use of linked data. It would have been preferable for the deferred survey to be administered at the same time interval for all trainings for the purposes of comparison and consistency.

This is the first delivery and evaluation of a tailored ethics program for people who are responsible for the review of HREC applications proposing to use linked data in Australia. This study contributes to the development of a robust ethical and legal training platform for HREC members and their staff, which is a necessary foundation for the conduct of ethically sound data linkage research in Australia.

## Endnotes

^a^National Health and Medical Research Centre. National Statement on Ethical Conduct of Research Involving Humans: Canberra; Australian Government; 2007. p105.

^b^A chairperson; lay person; person with knowledge of, and current experience in, the professional care, counselling or treatment of people; person who performs a pastoral care role in the community, lawyer, person with research experience.

## Abbreviations

HREC: Human research ethics committee
PHRN: Population health research network
NHMRC: National health and medical research centre
WA: Western australia
NSW: New south wales
SA: South australia
ACT: Australian capital territory
Tas: Tasmania
